# Associations between recreational cannabis legalization and cannabis use disorder treatment outcomes in California, 2010–2021

**DOI:** 10.1186/s42238-025-00323-6

**Published:** 2025-08-18

**Authors:** Brittany Bass, Howard Padwa, Dhruv Khurana, Darren Urada

**Affiliations:** Department of Addiction Psychiatry, Semel Institute for Neuroscience and Human Behavior, University of California, Los Angeles, 10911 Weyburn Avenue, Suite 200, Los Angeles, CA 90024 USA

**Keywords:** Cannabis, Recreational cannabis legalization, Cannabis use disorder treatment, Cannabis use disorder treatment retention

## Abstract

**Background:**

Despite extensive research on the broader effects of recreational cannabis legalization (RCL), limited attention has been given to its impact on cannabis use disorder (CUD) treatment outcomes. This study addresses this gap by analyzing CUD treatment outcomes, including 90-day treatment retention and successful discharge.

**Methods:**

Data were collected from a dataset of all publicly funded cannabis use disorder treatment delivered in California between January 2010 and December 2021 (*n* = 192,580). RCL’s impacts on CUD treatment outcomes was estimated using an individual-level pre-post time series model, including individual and county-level characteristics via logistic regression.

**Results:**

California’s RCL was associated with a significant decrease in the probability of 90-day treatment retention and also of successful discharge from treatment for patients with a CUD. RCL was also associated with a decrease in the probability of 90-day CUD treatment retention for adults ages 21 + and White Non-Hispanics, and a decrease in the probability of successful discharge for White Non-Hispanics, and an increase in the probability of successful discharge for males and adults ages 21+. We find no association between RCL and treatment retention or successful discharge for Black Non-Hispanic or Hispanic patients.

**Conclusions:**

The implementation of California’s RCL was associated with a decline in CUD treatment outcomes. The findings underscore the need for ongoing evaluation of cannabis legalization’s effects on public health to inform policy and practice in the context of evolving cannabis regulations.

**Supplementary Information:**

The online version contains supplementary material available at 10.1186/s42238-025-00323-6.

## Introduction

As of June 2024, 24 states plus the District of Columbia have legalized cannabis for recreational (i.e., adult) use (NCSL 2024). Recreational cannabis legalization (RCL) has been associated with increases in the prevalence of cannabis use (Cerda et al. 2020; Hall and Lynksey 2020; UNODC, 2022), more frequent cannabis use, increased prevalence of cannabis use disorder (CUD) among adults (Cerda et al. 2020). CUDs are serious health conditions that dramatically impact health and quality of life, and they are associated with increased risk for other substance use disorders (SUDs) and mental health conditions (Connor et al. 2021), so it is critical for public health to effectively address CUD as rates continue to rise in the age of legalization.

Previous research has shown that while RCLs are associated with increased CUD prevalence, they are also associated with decreased likelihood of admission to CUD treatment (Bass et al. 2024), meaning that increasing numbers of people who need treatment are not receiving it in jurisdictions that have implemented RCLs. In this paper, we expand on this work to examine if the effectiveness of CUD treatment has been impacted by RCLs. We examine RCLs’ impacts on 90-day treatment retention, which is associated with positive substance use disorder (SUD) treatment outcomes (NIDA 2018), and treatment completion, which is associated with sustained improvements in substance use behaviors, criminal justice, economic, and health domains following treatment (Riviera et al. 2021). Increased knowledge about RCLs’ impacts on CUD treatment effectiveness is needed to determine if current CUD treatment practices are responsive to the needs of patients where RCLs are in place, or if services need to adapt to treat CUDs more effectively in the age of legalization.

A priori, it is unclear in which direction we may expect RCLs to impact CUD treatment outcomes. For example, RCLs may negatively impact treatment outcomes if legalization normalizes and reduces the perceived risk (Hall and Lynskey 2016), which may reduce motivation to fully engage in and complete treatment. Alternatively, legalization may positively impact treatment outcomes if it reduces stigma (King et al. 2024; Wen et al. 2019) and increases the willingness to seek and engage in treatment (van der Pol et al. 2013). Therefore, we empirically assess the effect of RCL on CUD treatment outcomes using data on CUD treatment outcomes for individuals who received publicly funded CUD treatment in California from January 2010 through December 2021.

To our knowledge, only one other study has assessed the impacts of cannabis policies on CUD treatment outcomes. Bourdon et al. (2021) used national data from the Treatment Episode Data Set – Discharges from 2006 to 2017 to examine the impact of cannabis policies (decriminalization, medical, and recreational) and referral sources (criminal justice vs. voluntary) on treatment completion and length of stay, and found that cannabis policies have no impact on successful treatment completion, while decriminalization policies impede treatment engagement past 90 days. Though the scope of the current study is like that of Bourdon et al. (2021), our study differs and contributes to our understanding of cannabis policies and treatment outcomes in several ways.

First, our study focuses specifically on the impact of a recreational cannabis legalization policy in a single state (California) that had already decriminalized possession of small amounts of cannabis in 2010 (Senate Bill 1449) and implemented a diversion-to-treatment policy for certain non-violent drug offenses in 2000 (Proposition 36). As such, we can more carefully separate the role of RCL specifically on treatment outcomes, by isolating it from the effects of decriminalization and criminal justice reform (Bass et al. 2024). Second, unlike Bourdon et al. (2021), which only included data through 2017, our analysis includes data through the end of 2021. The effects of RCL on CUD treatment outcomes may have evolved between 2017 and 2021, due to increases in THC potency in states that have implemented RCLs in recent years (ElSholy et al. 2021; Firth et al. 2020; Hall and Lynskey 2020; Hasin et al. 2021; Smart et al. 2017). The availability of more potent cannabis products has been associated with quicker progression from cannabis use to CUD (Arterberry et al. 2019) and more severe symptoms of CUD (Freeman and Winstock 2015), thereby potentially influencing the outcomes of CUD treatment. Third, we assessed the impact of adult use legalization on treatment outcomes for multiple subgroups beyond referral source to uncover potential heterogeneity in treatment outcomes that may be masked in the aggregate.

## Materials and methods

This observational study used logistic regressions to determine associations between recreational cannabis legalization in California and CUD treatment outcomes among individuals who received publicly funded CUD treatment and were discharged from treatment in the state between January 2010 and December 2021 (*n* = 192,580). All data were collected from the California Outcomes Measurement System, Treatment (CalOMS-Tx), a reporting system for all publicly funded SUD treatment services delivered in California. To our knowledge, within cannabis research, CalOMS-Tx has only been used for cannabis research on the association between recreational cannabis legalization and CUD treatment admissions (Bass et al. 2024), and medical cannabis users in SUD treatment (Swartz 2010).

### Variables

#### Treatment variable

##### Recreational cannabis legalization (RCL)

On November 8, 2016, California voters passed Proposition 64, which legalized the personal use and cultivation of cannabis for adults 21 years of age or older (California Courts 2016). We created a binary variable equal to 1 for all patients discharged from treatment in or after November 2016 and 0 for patients discharged before. We based the treatment variable on the discharge date rather than the admission date for several reasons. First, the primary outcomes of interest were measured at discharge, not admission, and using the discharge date aligns the policy exposure (RCL) with the time when the outcome was observed. Second, the legalization of recreational cannabis may have influenced the SUD treatment environment over time. Specifically, it may have influenced patient attitudes and behaviors, provider practices, and referral patterns, changes that would unfold during treatment or by the time of discharge, not necessarily at the time of admission. Third, defining treatment based on the discharge date avoided the misclassification of RCL exposure. That is, if a patient was admitted before November 2016 but discharged after, they may have experienced treatment post-RCL exposure and classifying them as “pre-RCL” based on their admission date would misclassify their exposure to RCL’s effects.

#### Dependent variables

##### 90-day treatment retention

Patients were identified as having been in treatment for at least 90 consecutive days before discharge for CUD if they indicated marijuana/hashish as their “Primary Drug” in CalOMS-Tx as a proxy, and had a “time in treatment” for 90 days or more. Research has shown that to produce and maintain positive outcomes for SUD treatment patients, participation in treatment for at least 90 days is recommended (NIDA 2018). Following this research, time in treatment was determined by calculating the difference between the patient’s discharge date and their admission date in days. We created a binary variable equal to 1 if the patient was in treatment for CUD for 90 consecutive days or more, and equal to 0 if the patient was in treatment for CUD for less than 90 consecutive days for all outpatient and residential levels of care.

##### Successful discharge

Patients were identified as having been successfully discharged from CUD treatment if they indicated marijuana/hashish as their “Primary Drug” in CalOMS-Tx as a proxy, and their discharge status in CalOMS-Tx was coded as any one of the following: completed treatment and referred; completed treatment and not referred; left before completion with satisfactory progress (indicates the patient has not completed the first phase of treatment and is being referred to continue the same treatment service elsewhere, or is changing to a different treatment service (DHCS 2014); left before completion with satisfactory progress – administrative discharge (reported for a patient who made satisfactory progress in the treatment service, who did not complete the treatment service as planned (e.g., left early), and could not be located to receive a referral for further SUD treatment or to conduct a discharge interview (DHCS 2014). A similar definition of successful discharge (or “treatment completion”) is used in Wright (2025), who assessed racial disparities in outpatient SUD treatment completion in New York from 2004 to 2024. We created a binary variable equal to 1 if the patient’s discharge status was coded as any one of the following statuses listed above, and equal to 0 if the patient’s discharge status was coded as any one of the following: left before completion with unsatisfactory progress; left before completion with unsatisfactory progress – administrative discharge; death; incarceration.

#### Control variables

We included several individual-level covariates to control for individual-specific characteristics to help isolate the effect of recreational cannabis legalization from these other variables that may influence CUD treatment retention and successful discharge. In line with Andersen’s Behavioral Model of Health Services Utilization (Anderson 1995), we treated age at admission (continuous), race/ethnicity (White Non-Hispanic, Black Non-Hispanic, Hispanic, Other Non-Hispanic), sex, high school graduate status (yes/no), veteran status (yes/no) and ever diagnosed with a mental illness (yes/no) as predisposing factors; Medi-Cal beneficiary status (yes/no) and involvement in the criminal justice system at admission (yes/no) as enabling factors; and secondary drug (alcohol, cocaine/crack, heroin, marijuana, methamphetamine, other), and type of treatment service (outpatient or residential) as need factors. All individual-level covariates are taken directly from CalOMS-Tx and were patient-reported at admission.

We also included several county-level covariates at the month-year level (i.e. including data for each month and year of our sample period to account for time-varying characteristics), including the poverty rate and the unemployment rate (obtained from the American Community Survey for 2010–2021), indicators for whether a county had an adult or juvenile drug court operating in it (requested and obtained from the Judicial Council of California), the log number of adult and juvenile arrests (obtained from the Department of Justice’s Open Justice website), an indicator for participation in the Drug Medi-Cal Organized Delivery System (DMC-ODS) waiver (available from the CA Department of Health Care Services), a stay-at-home order indicator to capture impacts of COVID-19 (obtained from the University of Arizona’s Research on COVID-19 Interventions and Impacts Group), and indicators for criminal justice-related reform in California, including Proposition 36 (Changes to Three Strikes Sentencing Initiative, November 2012 (Legislative Analyst’s Office 2012)), Proposition 47 (also known as “The Safe Neighborhoods and Schools Act”, November 2014 (California Courts 2014)), and AB 109 (also known as the “California Public Safety Realignment Act”, October 2011 (California Department of Corrections and Rehabilitation 2013)). Our county-level covariates served as contextual enabling influences. These individual- and county-level covariates can influence both the likelihood of cannabis use and treatment outcomes independent of recreational legalization and must be controlled for so as not to spuriously attribute the impact of legalization to our outcomes of interest.

### Statistical methods

To determine the associations of recreational cannabis legalization with CUD treatment outcomes (90-day retention and successful discharge), we estimated an individual-level pre-post time series model, including rich individual and county-level characteristics via logistic regressions. We also estimated associations among demographic subgroups, including, males (relative to females), adults ages 21+ (relative to young adults ages 18–20), White Non-Hispanics (relative to all other races), Black Non-Hispanics (relative to all other races), and Hispanics (relative to Non-Hispanics) and each subgroup analysis includes an interaction term between the characteristic of interest (e.g., male) and RCL, plus the main effects for both variables. We can interpret the coefficients reported in Table 2 as the expected change in the probability of the CUD treatment outcome after RCL, compared to before the passage of RCL. All confidence intervals reported are two-sided 95% confidence intervals. This approach mirrored that of Bass et al. (2024), who estimated the association between RCL and CUD treatment admissions overall and for subgroups using CalOMS-Tx. We also conducted sensitivity analyses using alternate model specifications (see Supplemental Table 1). Analyses were conducted using Stata version 17.

### Human subjects protections

The University of California, Los Angeles Office of Human Research Protection Program reviewed this project and determined that it did not need institutional review board review or certification of exemption.

## Results

See Table 1 for an overview of the study sample by pre- and post-RCL passage. Among our outcomes of interest, of the 192,580 patients receiving treatment for CUD, 50% remained in treatment for at least 90 days before RCL implementation, compared to only 40% after. Similarly, 63% of patients were successfully discharged pre-RCL, versus 53% post-RCL. The distributions among individual and county-level covariates remain fairly stable across both periods, with the exception of Medi-Cal beneficiary status and mental illness diagnosis, both of which increase post-RCL.

**Table 1:** Descriptive statistics of analysis variables, January 2010 - December 2021

	Pre - RCL (January 2010 - October 2016)		Post - RCL (November 2016 - December 2021)		*p*-value
	Proportion (Mean)	Standard Deviation	Proportion (Mean)	Standard Deviation	
	(1)	(2)	(3)	(4)	(5)
**Outcomes**					
90-day Treatment Retention	0.50	0.50	0.40	0.49	0.00
Successful Discharge	0.63	0.48	0.53	0.50	0.00
**Individual-level covariates**					
Involved in CJ system	0.52	0.50	0.49	0.50	0.00
High school graduate	0.32	0.47	0.42	0.49	0.00
Male	0.71	0.46	0.68	0.47	0.00
Age at admission	22.64	10.18	24.30	10.64	0.00
Medi-cal beneficiary	0.55	0.50	0.77	0.42	0.00
Mental illness diagnosis	0.17	0.38	0.28	0.45	0.00
Veteran status	0.01	0.11	0.01	0.11	0.05
Outpatient treatment	0.89	0.31	0.86	0.35	
Race					
White Non-Hispanic	0.23	0.42	0.21	0.41	0.00
Black Non-Hispanic	0.17	0.37	0.14	0.35	0.00
Hispanic	0.53	0.50	0.57	0.50	0.00
Other Non-Hispanic	0.08	0.27	0.08	0.27	0.00
Secondary Drug					
Alcohol	0.33	0.47	0.22	0.42	0.00
Cocaine/Crack	0.03	0.17	0.03	0.17	0.43
Heroin	0.01	0.08	0.01	0.08	0.08
Marijuana	0.01	0.09	0.01	0.10	0.00
Methamphetamine	0.14	0.34	0.16	0.37	0.00
None	0.45	0.50	0.52	0.50	0.00
Other	0.04	0.19	0.05	0.21	0.00
**County-level covariates**					
Poverty Rate	0.17	0.05	0.14	0.04	0.00
Unemployment rate	0.10	0.04	0.06	0.03	0.00
Adult drug court	0.94	0.24	0.92	0.28	0.00
Juvenile drug court	0.51	0.50	0.37	0.48	0.00
# Juvenile arrests	10,062.01	11,396.27	2,345.91	2,288.40	0.00
# Adult arrests	104,537.10	114,764.90	60,260.09	66,179.67	0.00
DMC-ODS waiver	0.00	0.00	0.63	0.48	0.00
Stay at home order	0.00	0.00	0.12	0.32	0.00
Prop 36	0.52	0.50	1.00	0.00	0.00
Prop 47	0.19	0.40	1.00	0.00	0.00
AB 109	0.73	0.44	1.00	0.00	0.00
*Observations*	*138*,*566*		*54*,*014*		

Notes: Proportions are presented for binary outcomes and covariates (90-day Treatment Retention, Successful Discharge, involved in the criminal justice system, high school graduate, male, Medi-cal beneficiary, mental illness diagnosis, veteran status, outpatient treatment, race categories, secondary drug categories, adult and juvenile drug court, DMC-ODS waiver, stay at home order, Prop 36, Prop 47, and AB 109), and means are presented for continuous covariates (age at admission, poverty rate, unemployment rate, and # of juvenile and adult arrests). The p-value presented in column (5) is from a two-sample t-test of columns (1) and (3)

**Table 2:** Associations between RCL and CUD treatment outcomes

	Overall CUD Discharges	Male	Adult 21+	White Non-Hispanic	Black Non-Hispanic	Hispanic
	(1)	(2)	(3)	(4)	(5)	(6)
**Panel I: 90-day Treatment Retention**						
***RCL***	-0.183***	0.008	-0.013***	-0.029***	-0.004	-0.003
	(0.006)	(0.005)	(0.005)	(0.007)	(0.005)	(0.006)
	[-0.194,-0.172]	[-0.003,0.018]	[-0.023,-0.003]	[-0.043,-0.015]	[-0.014,0.006]	[-0.016,0.009]
*Observations*	192,580	192,580	192,580	192,580	192,580	192,580
**Panel II: Successful Discharge**						
***RCL***	-0.041***	0.017***	0.013**	-0.038***	-0.004	0.006
	(0.006)	(0.005)	(0.005)	(0.007)	(0.005)	(0.006)
	[-0.053,-0.030]	[0.006,0.027]	[0.003,0.023]	[-0.052,-0.023]	[-0.014,0.006]	[-0.006,0.018]
*Observations*	163,308	163,308	163,308	163,308	163,308	163,308

Notes: Average marginal effects from logistic regressions are presented. Standard errors are reported in parentheses, and 95% confidence intervals are reported in brackets. Each column presents a separate regression. All regressions control for all individual-level covariates at admission, and county-level covariates, and county and year fixed effects, as discussed in the text. Regressions in columns (2)–(6) present subgroup analyses, and each regression includes an interaction term between the characteristic and RCL, plus the main effects for both variables. *** represents statistical significance at the 1% level, ** represents statistical significance at the 5% level, and * represents significance at the 10% level

Table 2 shows the average marginal effects (AME) from logistic regressions of RCL’s association with CUD treatment outcomes. Each column presents a separate regression. Panel I shows the results for 90-day treatment retention, and Panel II shows the results for successful discharge.

Panel I Column (1) of Table 2 suggests that after the passage of RCL in California, the probability of remaining in CUD treatment for at least 90 days significantly decreased by 18.3 percentage points (AME = -0.183; 95% CI -0.194 to -0.172) after adjusting for individual and county-level covariates. In subgroup analyses, we found that RCL was associated with a decrease in the probability of remaining in CUD treatment for at least 90 days for adults ages 21+ (AME = -0.013; 95% CI -0.023 to -0.003), and White Non-Hispanics (AME = -0.029, 95% CI-0.043 to -0.015). However, we found no association between the passage of RCL and the probability of remaining in treatment for at least 90 days for males, Black Non-Hispanic, and Hispanic patients.

Panel II Column (I) of Table 2 suggests that after the passage of RCL, the probability of successful discharge decreased by 4.1 percentage points (AME = -0.041, 95% CI -0.053 to -0.030). Subgroup results showed that RCL was associated with an increase in the probability of successful discharge for males (AME = 0.017, 95% CI 0.006 to 0.027) and adults ages 21+ (AME = 0.013, 95% CI 0.003 to 0.023), and a decrease in the probability of successful discharge for White Non-Hispanics (AME = -0.038, 95% CI -0.052 to -0.023). We found no association between the passage of RCL and the probability of successful discharge for Black Non-Hispanic or Hispanic patients.

We next explored the sensitivity of our results to several different model specifications. In Supplemental Table 1, we present logistic regression estimates without individual-level, and county-level covariates, estimates from ordinary least squares (OLS) models, and logistic regression estimates for successful discharge, where now patients were identified as having been successfully discharged from CUD treatment if they indicated marijuana/hashish as their “Primary Drug” in CalOMS-Tx as a proxy, and their discharge status in CalOMS-Tx was coded as any one of the following: completed treatment and referred; completed treatment and not referred; left before completion with satisfactory progress. The robustness of the results presented in Table 2 is confirmed by Supplemental Table 1, with the results from all three specifications being quantitatively similar to those presented in Table 2.

## Discussion

This study explored the association between recreational cannabis legalization in California and CUD treatment outcomes using data on all publicly funded cannabis use disorder treatment delivered in California between January 2010 and December 2021 and an individual-level pre-post time series model. The findings of this study suggest that RCL in California decreased the overall probability of 90-day treatment retention and successful discharge for CUD patients. We also found that RCL decreased the probability of 90-day treatment retention for adults ages 21 + and White Non-Hispanics, increased the probability of successful discharge for males and adults ages 21+, and decreased the probability of successful discharge for White Non-Hispanics. The decrease in the probability of 90-day treatment retention and successful discharge for CUD patients is problematic since legalization is associated with increased prevalence of CUD (Cerda et al. 2020), highlighting the need for more effective treatment. The decrease in 90-day treatment retention found in the current study aligns with that of Bourdon et al. (2021), suggesting that adult use legalization is associated with worsening CUD treatment outcomes. This may be the case for several reasons.

First, the passage of RCL may have normalized the use of cannabis and the perception that cannabis is now less harmful (Hall and Lynskey 2016), thereby making it difficult for patients to remain motivated to reduce their cannabis use while in treatment and remain engaged in treatment. Additionally, recent evidence suggests that referrals to CUD treatment decreased after recreational cannabis legalization in the US (Mennis et al. 2023). This may be particularly true for White Non-Hispanic patients, who, on average, may have more access to legal cannabis markets post-legalization (Harris and Martin 2021). This access may have led to greater normalization and social acceptance of use, a lowering of the perceived severity of CUD, and potentially less social and legal pressure to remain engaged in treatment relative to minority populations who have historically been subjected to stricter cannabis enforcement (ACLU 2020; Alexander 2021; Taxy et al. 2015).

Second, it could be the case that traditional CUD treatment methods, such as motivational enhancement therapy and cognitive behavioral therapy (Sherman and McRae-Clark 2016), may not adequately address new challenges presented by highly potent cannabis products emerging post-legalization or changing societal attitudes, potentially reducing treatment retention and successful discharge. There are currently no behavioral or pharmacological treatments that have been proven effective in treating CUD (Connor et al. 2021; Kondo et al. 2020; Patel 2022), and worsening CUD treatment outcomes under legalization underscore the need to develop, test, and implement evidence-based CUD treatment interventions. Lastly, with recreational legalization, there may be shifts in healthcare resources and priorities as admissions to treatment for CUD have decreased (Bass et al. 2024), potentially affecting the availability or quality of CUD treatment services. Taken together, these factors can contribute to a decrease in treatment retention and successful discharge for cannabis use disorder following legalization, highlighting the complex interaction between policy changes, societal attitudes, and treatment outcomes, though further research is needed to verify this.

Though the overall results for CUD patients suggest a negative association between RCL and CUD treatment outcomes, we do find a positive association between RCL and successful discharge for males and adults aged 21+. Regarding adults ages 21+, since they can use cannabis legally, their treatment goals may shift in a post-legalization landscape. Adults may choose shorter treatment durations or less intensive treatment, which could lead to earlier discharge from treatment, but with the treatment considered “successful” by the provider under potential new treatment models and flexibility. Similarly, for males, who have higher rates of CUD relative to females (Cuttler et al. 2016), their treatment goals may also shift post-legalization due to less stigma and the normalization of cannabis use and may be more motivated to complete treatment to maintain functional stability (e.g., work, family).

The findings of this study highlight a critical need for CUD treatment policies that effectively address the complex and evolving impacts of recreational cannabis legalization on treatment outcomes. California’s RCL was associated with declines in treatment retention and successful discharge, especially among adults aged 21 and older and White Non-Hispanic populations, emphasizing the urgency of developing more effective, evidence-based, and tailored treatment strategies. Policy efforts should prioritize the creation and funding of interventions that reflect shifting societal attitudes and the growing normalization of cannabis use, ensuring that treatment programs remain engaging, relevant, and capable of sustaining patient motivation.

## Limitations

Though this study adds to our understanding of the impacts of cannabis legalization on substance use disorder treatment outcomes, it is not without its limitations. First, although an SUD diagnosis is required for a patient to receive publicly-funded treatment services, and patients admitted to treatment with cannabis as a primary drug is a strong indicator of CUD, formal documentation of a CUD in CalOMS-Tx is lacking. Second, the study’s analysis is focused solely on California and is simply a pre-post analysis and did not yield a causal estimate of the impact of RCL on CUD outcomes, and the results should be interpreted with caution. Ideally, a difference-in-difference analysis would be most appropriate, but administrative data similar to CalOMS-Tx, which includes both admissions and discharge records for the same individual over time, for another state (or states) is publicly unavailable. Additionally, the generalizability of our findings to other states or time periods outside of our analysis period may be challenged since we only examine the impacts of RCL on CUD treatment in California. Third, due to a lack of data availability, we are unable to test the potential mechanisms discussed above. Future work should aim to uncover if these mechanisms are at play and affect treatment retention and successful discharge. Nevertheless, the associative findings of this study are important for policymakers in other states and countries considering legalizing adult-use cannabis, as there is limited evidence of the impacts of RCL on CUD treatment outcomes.

## Conclusions

Our findings suggest that recreational cannabis legalization in California is associated with a decline in treatment outcomes for cannabis use disorder. Exploring the specific mechanisms through which legalization may affect CUD treatment outcomes is crucial, and future work should assess factors such as changes in access to treatment and treatment methods, shifts in public perception of cannabis use, and variations in regulatory frameworks, which could all influence the effectiveness of CUD treatment. As more states and countries enact recreational cannabis legalization, comprehensive, causal research will be essential for informing evidence-based public health policies.

## Electronic supplementary material

Below is the link to the electronic supplementary material.

Supplementary material 1

## Data Availability

The CalOMS-Tx data that support the findings of this study are available from the California Department of Health Care Services, but restrictions apply to the availability of these data, which were used under license for the current study, and so are not publicly available. Data on the control variables used in this study are available from the authors upon request.
