# Supplementary material for: Associations between recreational cannabis legalization and cannabis use disorder treatment outcomes in California, 2010–2021
Source: J Cannabis Res. 2025 Aug 18;7:60. doi: 10.1186/s42238-025-00323-6 (PMC12360006; doi:10.1186/s42238-025-00323-6)
Supplement: Supplementary file 1 — Supplementary material 1 [file 42238_2025_323_MOESM1_ESM.docx]

| **Supplemental Table 1: Associations between RCL and CUD Treatment Outcomes - Sensitivity Analyses** | | | | | | |
| --- | --- | --- | --- | --- | --- | --- |
|  | **Overall CUD Discharges** | **Male** | **Adult 21+** | **White Non-Hispanic** | **Black Non-Hispanic** | **Hispanic** |
|  | (1) | (2) | (3) | (4) | (5) | (6) |
| ***Panel I: No covariates*** | | | | | | |
| **90-day Treatment Retention** | | | | | | |
| ***RCL*** | -0.083*** | 0.012** | -0.011** | -0.041*** | 0.000 | -0.003 |
| *Avg. Marginal Effect* | (0.003) | (0.005) | (0.005) | (0.007) | (0.005) | (0.006) |
|  | [-0.088,-0.078] | [0.002,0.023] | [-0.021,-0.001] | [-0.055,-0.026] | [-0.010,0.010] | [-0.015,0.009] |
| *Observations* | 192,580 | 192,580 | 192,580 | 192,580 | 192,580 | 192,580 |
| **Successful Discharge** | | | | | | |
| ***RCL*** | -0.112*** | 0.017*** | 0.008 | -0.048*** | -0.003 | 0.011* |
| *Avg. Marginal Effect* | (0.003) | (0.006) | (0.005) | (0.007) | (0.005) | (0.006) |
|  | [-0.117,-0.107] | [0.006,0.028] | [-0.002,0.019] | [-0.063,-0.034] | [-0.013,0.007] | [-0.001,0.024] |
| *Observations* | 163,308 | 163,308 | 163,308 | 163,308 | 163,308 | 163,308 |
| ***Panel II: Ordinary Least Squares (Beta coefficients)*** | | | | | | |
| **90-day Treatment Retention** | | | | | | |
| ***RCL*** | -0.182*** | 0.007 | -0.007 | -0.024*** | -0.007 | -0.001 |
|  | (0.006) | (0.005) | (0.005) | (0.007) | (0.005) | (0.006) |
|  | [-0.193,-0.171] | [-0.003,0.017] | [-0.017,0.003] | [-0.037,-0.011] | [-0.017,0.002] | [-0.013,0.011] |
| *Observations* | 192,580 | 192,580 | 192,580 | 192,580 | 192,580 | 192,580 |
| **Successful Discharge** | | | | | | |
| ***RCL*** | -0.041*** | 0.012** | 0.015*** | -0.035*** | -0.008 | 0.01 |
|  | (0.006) | (0.006) | (0.005) | (0.007) | (0.005) | (0.006) |
|  | [-0.053,-0.030] | [0.002,0.023] | [0.004,0.025] | [-0.050,-0.021] | [-0.019,0.002] | [-0.002,0.023] |
| *Observations* | 163,308 | 163,308 | 163,308 | 163,308 | 163,308 | 163,308 |
| ***Panel III: Recode of Successful Discharge*** | | | | | | |
| **Successful Discharge** | | | | | | |
| ***RCL*** | -0.056*** | 0.010* | 0.003 | -0.025*** | 0.007 | -0.007 |
| *Avg. Marginal Effect* | (0.006) | (0.006) | (0.005) | (0.008) | (0.005) | (0.006) |
|  | [-0.067,-0.044] | [-0.001,0.021] | [-0.007,0.014] | [-0.039,-0.010] | [-0.003,0.017] | [-0.020,0.005] |
| *Observations* | 163,308 | 163,308 | 163,308 | 163,308 | 163,308 | 163,308 |
| Notes: Standard errors are reported in parentheses, and 95% confidence intervals are reported in brackets. Each column presents a separate regression. All regressions in Panels II and III control for all individual-level covariates at admission, and county-level covariates, and county fixed effects, as discussed in the text. Regressions in columns (2)–(6) present subgroup analyses, and each regression includes an interaction term between the characteristic and RCL, plus the main effects for both variables. In Panel III, successful discharge is a binary variables = 1 if patients indicated marijuana/hashish as their “Primary Drug” in CalOMS-Tx as a proxy, and their discharge status in CalOMS-Tx was coded as any one of the following: completed treatment and referred; completed treatment and not referred; left before completion with satisfactory progress. *** represents statistical significance at the 1% level, ** represents statistical significance at the 5% level, and * represents significance at the 10% level. | | | | | | |
